# MURCS Association with Partial Duplication of the Distal Long Chromosome 5 and Unilateral Ovarian Agenesis

**DOI:** 10.1155/2013/105052

**Published:** 2013-02-17

**Authors:** Anna Dabkowska-Huc, Piotr Skalba, Antoni Pyrkosz

**Affiliations:** ^1^Department of Gynecological Endocrinology, Medical University of Silesia, Medykow 14, 40-752 Katowice, Poland; ^2^Department of General and Molecular Biology and Genetics, Medical University of Silesia, Medykow 18, 40-752 Katowice, Poland

## Abstract

A combination of the congenital abnormalities, Müllerian duct aplasia, renal aplasia, and cervicothoracic somite dysplasia, is defined as the MURCS association. Various genetic defects have been described in the MURCS association so far, yet the unambiguous molecular basis of these disorders has not been established. We report the case of an 18-year-old woman who presented with primary amenorrhea, right kidney, Arnold-Chiari malformation, and Klippel-Feil syndrome. In addition, the patient showed the following unusual features: right ovarian and Skenes gland agenesis, cubitus valgus with hyperextension and decreased range of motion at elbows, and facial changes. Moreover, the performed DNA analysis showed interstitial duplication in chromosome 5 (5q35.1). In the duplicated region, there are genes whose function is not well known. It is thought that they have an influence on the early stages of development and their joining in the later period can lead to neoplastic disorders, especially leukemias.

## 1. Background

MURCS association is the combined occurrence of the following disorders: Müllerian duct aplasia, renal aplasia, and cervicothoracic somite dysplasia. Cervicothoracic somite dysplasia is described in Klippel-Feil syndrome (KFS). Changes in the relations in the base of the neck and head can lead to the development of Arnold-Chiari malformation (ACM). ACM is a congenital malformation of the central nervous system, traditionally defined as downward herniation of the cerebellar tonsils through the foramen magnum [1]. 

In our case, in addition to the typical features, the following abnormalities are unusual for MURCS association: right ovarian and Skenes gland agenesis, cubitus valgus with hyperextension and decreased range of motion at elbows, and changes in facial appearance [2–5]. MURCS girls are karyotypically female (46, XX). Various genetic defects have been described in the MURCS association so far, yet the unambiguous molecular basis of these disorders has not been established [6, 7]. The relationship of 5q35.1 duplication with characteristic features for the MURCS association is interesting. In the accessible literature, we have not found the connection of this chromosomal duplication of 5q35.1 with MURCS association. 

## 2. Case History

**Figure 1:**
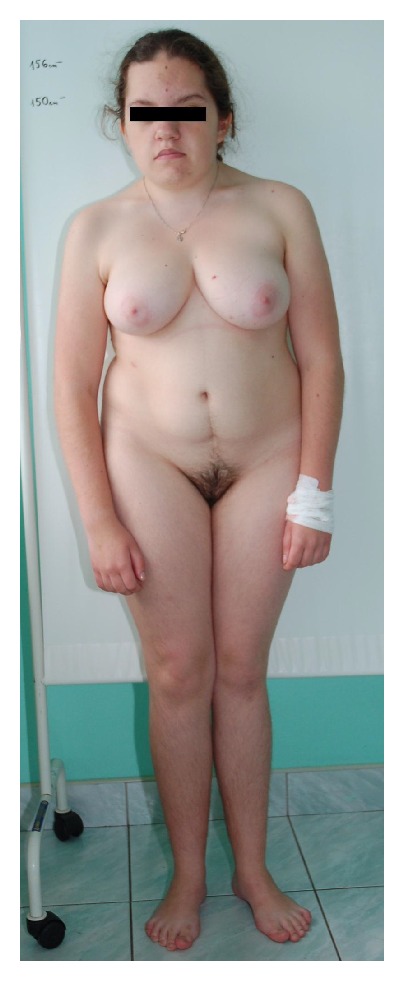
The phenotype of the patient.

The 18-year-old patient was admitted to the Gynecological Endocrinology Department because of primary amenorrhea (Figure 1).

Her height was 1.56 m, weight 66 kg, BMI 27 kg/m^2^, and occipitofrontal head circumference 59 cm (>97th percentile). She was born at term with hypotrophy. The facial features consisted of a frontal bossing, a long and full face, with middle part hypoplasia, and secondary prognathism, depressed and broad nasal root, with long and fleshy nose, long philtrum, thick lips, low-set ear, and large ear lobes. Palate is high and narrow. Neck is broad and short, secondary to cervical vertebral defects, with low hair line. Cervicothoracic vertebral defects (Klippel-Feil malformation) and Sprengel scapular anomaly with probable torticollis in the infantile period, were evident and induced a little face and thoracic asymmetry. Thoracolumbar scoliosis with excessive lumbar lordosis is visible. Trunk is clearly shorter than legs. Valgus elbows with hyperextension and decreased range of motion. Valgus knee, small feet with sandal gap and fifth toe clinodactyly; nails are hypoplastic. 

Arnold-Chiari malformation type 1 was diagnosed in the patient in the prenatal period. Klippel-Feil syndrome was additionally diagnosed in the period of childhood.

Examination and psychological testing showed average mental development. The patient in addition to defects in the cervical spine also shows abnormalities of the spine in the thoracic and lumbar segments. The patient in childhood was found to have right kidney agenesis and a displaced left kidney on the left side of the bladder with a wide, short ureter. Ureteral plastic surgery was performed at 2 years of age.

The physical examination revealed normal external genital and breast development and normal development of axillary and pubic hair. 

Gynecological examination showed oversized vaginal labia which hid the entrance to the vestibule of the vagina. There was no entrance to the Skenes gland on the right side. There was a complete vaginal and uterus atresia. Lack of the uterus and the right ovary was confirmed by ultrasound and magnetic resonance imaging.

In the ultrasound examination, ectopic left kidney located on the left side of the bladder was found. Centrifugally in relation to the left kidney was an ovary with one dominant follicle and several small follicles. Urography confirmed the normal secretory function of the left kidney and lack of a right kidney.

Blood levels of ovarian and pituitary hormones were mostly correct. 

**Figure 2:**
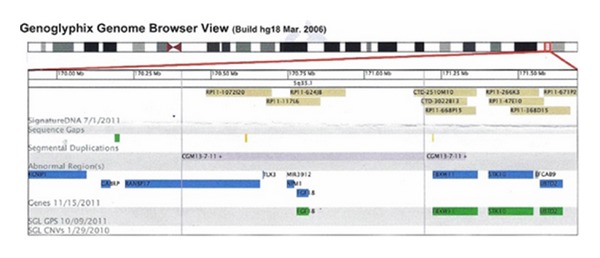
The genes located in the region of 5q35.1.

The examination of karyotype with the GTG method showed a normal female karyotype. Because of existing developmental disorders, we performed DNA analysis using CGH microarray. Our results showed karyotype 46, XX, dup (5q35.1), interstitial duplication in chromosome 5 containing 914.2-973.3 kb (genome position HG18: 170405455-171328275). In the critical region by OMIM, there are four genes—*RANBP17*,* TLX3*,* NPM1*,and* FGF18* and another gene *MIR3912* (Figure 2). The examination was performed using 135k NimbleGen CGX-12 chips.

In the duplicated region, including <1 Mb, there are genes whose function is not sufficiently known. Other anomalies are associated with the duplication of the region 5q. One of them is a Boston type craniosynostosis. In 2005, Hunter et al. [8] describe the characteristics of the Hunter-McdAlpine syndrome associated with the duplication of the region 5q35-qter. In our case, we have not identified those characteristics.

The knowledge about them results mainly from the comparison of conservative regions and observations on animal models. It is thought that they have an influence on the early stages of development and their joining in the later period may lead to neoplastic disorders (Table 1).

Based on the above, on clinical judgment, the MURCS association with unique chromosomal rearrangement—duplication of chromosome 5q35.1—was diagnosed.

## 3. Discussion

The MURCS association is remarkably variable and undoubtedly causally heterogeneous. One must verify various chromosomal imbalances, and therefore a chromosome analysis, especially using the array-CGH method, is appropriate in most cases. In addition to the broad variability of MURCS association, diagnosis may also be complicated by the existence of several syndromes with overlapping features.

Oppelt et al. [6] reviewed 521 cases from the literature and found 12 with MURCS association. Its incidence is 1 case per 50,000 women [9]. Some MURCS association cases belong to the malformation spectrum of DiGeorge phenotype [10, 11]. Hofstetter et al. [12] diagnosed a 16-year-old patient showing the cardinal features of MURCS association accompanied by a persistent left superior vena cava and atrial septal defect, orofacial clefting, and mild reduction deformities of the left hand.

Guerrier et al. [13] tried to find a candidate gene for Mayer-Rokitansky-Küster-Hauser syndrome, but they did not divide the genes typical for MURCS. They explored the gene for galactose-1-phosphate uridyl transferase (GALT), the gene encoding the CFTR chloride channel, genes acting during early development such as WT1 and PAX2, genes of anti-Müllerian hormone or its receptor, and the hepatocyte nuclear factor (HNF)-1*β* gene [14, 15]. But none showed any mutation and/or polymorphism associated with Müllerian aplasia.

The molecular basis of MURCS association remains unknown. However, the molecular analysis of the similar disorder Mayer-Rokitansky-Kuster-Hauser syndrome made by Ledig et al. revealed the candidate genes for this anomaly [16]. 

In our patient, interstitial duplication in chromosome 5 (5q35.1) was diagnosed. This mutation was not connected with MURCS association previously. In the accessible literature, we found a description of duplication in the region 5q35.1 connected with the occurrence of the developmental disorders holoprosencephaly and preaxial polydactyly [17]. 

It is important to underline that the technique applied by us does not show which gene has the decisive influence on the phenotype observed in our patient.

Moreover, in our patient with the duplication of 5q35.1, gain-of-function genes exist in the critical region (partial trisomy), not loss of function as in the above example. 

The combination of abnormal ovarian development and MURCS association occurs extremely rarely. Al Kaissi et al. [11] reported a 17-year-old girl with MURCS association and ovarian dysplasia. Tan et al. [18] described a patient with bicornuate uterus, right ovarian agenesis, unilateral multicystic dysplastic kidneys, and other birth defects not typically associated with MURCS. The present case deserves attention because of many atypical findings and unparalleled chromosomal rearrangement. However, the association between these conditions may be coincidental, and at present, these types of ovarian pathology are not considered to be part of the MURCS clinical spectrum.

## 4. Conclusions

MURCS is a rare congenital disorder, and therefore difficult to diagnose. This case deserves attention because it also presents unusual abnormalities and previously unreported interstitial duplication of chromosome 5q35.1, which may be associated with certain symptoms observed in the described patient.

The patient has given consent for the case report to be published.

## Figures and Tables

**Table 1:** Action of genes in region 5q35.1. Based on OMIM 20/11/2012.

Genes in region 5q35.1	Action of genes
*TLX3 *	T-cell leukemia is caused by the defect of the gene
*RANBP17 *	Acute lymphoblastic leukemia is caused by the mutation of the gene
*NPM1* and *ALK *	Fusion of these 2 genes can cause acute medullary leukemia
*FGF18 *	Participates in oncogenesis; it also influences the differentiation of osteoblasts and chondrocytes and the development of cartilage forming from mesenchymal lung tissue
